# Supplying a pharmacy for NASA exploration spaceflight: challenges and current understanding

**DOI:** 10.1038/s41526-019-0075-2

**Published:** 2019-06-13

**Authors:** Rebecca S. Blue, Tina M. Bayuse, Vernie R. Daniels, Virginia E. Wotring, Rahul Suresh, Robert A. Mulcahy, Erik L. Antonsen

**Affiliations:** 1 Aerospace Medicine and Vestibular Research Laboratory, The Mayo Clinic Arizona, Scottsdale, AZ 85054 USA; 2 GeoControl Systems, Inc, Houston, TX 77058 USA; 3 KBR, Houston, TX 77058 USA; 4 Department of Pharmacology and Chemical Biology and Center for Space Medicine, Baylor College of Medicine, Houston, TX 77030 USA; 5 Department of Preventive Medicine and Community Health, University of Texas Medical Branch, Galveston, TX 77555-1110 USA; 6 National Aeronautics and Space Administration (NASA), Johnson Space Center, Houston, TX 77058 USA; 7 Department of Emergency Medicine and Center for Space Medicine, Baylor College of Medicine, Houston, TX 77030 USA

**Keywords:** Preventive medicine, Pharmacokinetics, Pharmacodynamics, Clinical pharmacology

## Abstract

In order to maintain crew health and performance during long-duration spaceflight outside of low-Earth orbit, NASA and its international partners must be capable of providing a safe and effective pharmacy. Given few directed studies of pharmaceuticals in the space environment, it is difficult to characterize pharmaceutical effectiveness or stability during spaceflight; this in turn makes it challenging to select an appropriate formulary for exploration. Here, we present the current state of literature regarding pharmaceutical stability, metabolism, and effectiveness during spaceflight. In particular, we have attempted to highlight the gaps in current knowledge and the difficulties in translating terrestrial-based drug studies to a meaningful interpretation of drug stability, safety, and effectiveness in space. We hope to identify high-yield opportunities for future research that might better define and mitigate pharmaceutical risk for exploration missions.

## Introduction

In order to maintain crew health and performance during long-duration spaceflight outside of low-Earth orbit (LEO), the National Aeronautics and Space Administration (NASA) and its international partners must be capable of providing a safe, effective, and comprehensive pharmacy. Long-duration exploration missions will differ from missions in LEO in important ways, including extended mission durations, lack of resupply capability, prolonged exposure to the space radiation environment, and the absence of emergency medical return capability. These differences drive additional requirements for an exploration pharmacy: it must be stable for the duration of a given reference mission, predictably effective, and personalized to the crew and the unique physiological and psychological challenges they may face. Currently, we do not understand the effects of spaceflight on medication stability and effectiveness well enough to address these additional requirements. There have been few studies to date that effectively evaluate pharmaceutical parameters within the spaceflight environment, such as medication stability or effectiveness. In addition, a lack of clearly defined requirements to track medication use or response has hampered effective data collection. As a result, it is difficult to characterize pharmaceutical effectiveness or stability during spaceflight; this in turn makes it challenging to select an appropriate formulary for exploration.

Currently, NASA lacks consistent, comprehensive clinical evidence of pharmaceutical use and effects on the International Space Station (ISS). Anecdotal evidence suggests that medications might degrade more quickly in space than terrestrially, but there is insufficient evidence to characterize stability for long-duration spaceflight. Given known physiological changes associated with spaceflight, including effects on immune function, fluid shifts, and metabolism, it is likely that pharmacokinetics and pharmacodynamics (PK/PD) may be altered in space, though there is insufficient evidence to characterize this risk. Exploration spaceflight may also drive the need for technological advances that enhance chemical pharmaceutical stability, allow for in-flight analysis of drug safety and effectiveness, and avoid excessive crew time commitment for consumable tracking and formulary maintenance all while minimizing the pharmaceutical footprint in both vehicle design and crewmember operations.

Here, we seek to present the current state of literature regarding pharmaceutical stability, metabolism, and effectiveness during spaceflight. In particular, we have attempted to highlight the gaps in current knowledge and the difficulties in translating terrestrial-based drug studies to a meaningful interpretation of drug response in space. We hope to identify high-yield opportunities for future research that might better define and mitigate pharmaceutical risk for exploration missions.

## Medication use and performance during spaceflight

Medication use during spaceflight is not comprehensively monitored, largely due to conflicting crew time demands and a desire to avoid onerous tracking procedures for common medication use. Crewmembers are allowed to take certain medications without first discussing with their flight surgeon, especially for common ailments, such as congestion or headache. In particular, over-the-counter medication use is rarely recorded. While astronauts are encouraged to discuss symptoms, illness, or medication use with their flight surgeon, much of this discussion occurs without required documentation. Private Medical Conferences (PMCs) between astronauts and their flight surgeons occur regularly throughout a mission, and flight surgeons can document illness or medication use in flight logs or the electronic medical record (EMR) following these conferences. This documentation is highly variable, physician-dependent, relies on crewmember recall potentially several days after medication use, and often lacks information, such as specific medications consumed, dosage, indication, effectiveness, or any side effects. The need for careful inventory tracking or monitoring of drug consumption has been a low priority due to the historical capability to frequently resupply medication stock.^1^ As a result, there is a paucity of information available regarding the frequency of medication consumption, the quantity of medications used over time, or the mass and volume of pharmaceuticals that might be needed for a given mission.

**Fig. 1:**
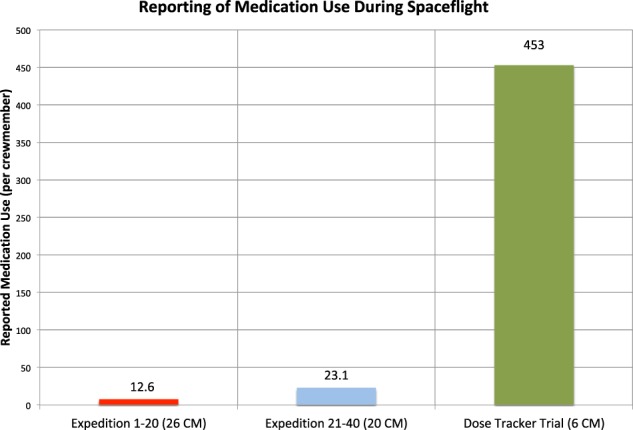
Reported use of medication per crewmember during ISS Expeditions 1–20, 21–40, and during the experimental Dose Tracker trial.^2,3^ Expeditions 1–20 had minimal requirements regarding reporting of medication use and relied on crewmember volunteering of data; Expeditions 21–40 provided slightly more guidance for flight surgeons in soliciting medication use information during Private Medical Conferences, but relied on crewmember recall. The Dose Tracker experiment employed medication-tracking technology that prompted crewmembers to report usage data, side effects, effectiveness, and symptom relief for each use of medication during flight. The experiment demonstrated the extensive amount of drug-use data lost by traditional medication use tracking. CM crewmembers

This data loss is well visualized by comparing documented medication use across historical ISS Expeditions. During Expeditions 1–20, medication usage reporting was not standardized and relied upon crewmember volunteering of information and flight surgeon documentation of usage in flight logs or the EMR. Twenty-six U.S. crewmembers flew to the ISS during these 20 expeditions; on average, documented usage indicated 12.6 medication doses taken per crewmember per mission.^2^ In subsequent expeditions, medication reporting was slightly more standardized. During PMCs for Expeditions 21–40, crewmembers were specifically queried on medication use for documentation by flight surgeons. While this relied upon crew recall, there was an attempt to more carefully track drug consumption during flight. During Expeditions 21–40, in total 20 crewmembers reported an average of 23.1 medications per crewmember over the course of a mission, nearly twice the average reported by the earlier expeditions (Fig. 1).^2^

Even so, there seems to be significant underreporting of medication usage, despite the more stringent documentation requirements. In 2017, the Dose Tracker experiment monitored pharmaceutical use by six crewmembers during their 5–6 month missions to the ISS (though data were only comprehensively recorded on five of the six study participants).^3,4^ During the study, an average of 453 medication uses were reported per crewmember during their missions, approximately four medications per crewmember per week.^2,3^ While the study continues to undergo analysis, early conclusions indicate that the participating crewmembers are not necessarily using more medications than prior astronauts but, rather, that medication usage has been grossly underreported in medical documentation.^2,3^

The reliance upon medication use should not be surprising. Given the role of screening and prevention, and the limitations of mass and volume on any space vehicle, the vast majority of medical conditions encountered during spaceflight tend to be managed by pharmaceutical intervention rather than invasive techniques. In general, the aerospace medical community has relied on medications to manage most medical concerns and works under the assumption that medications will be as effective during spaceflight as they are terrestrially.

**Fig. 2:**
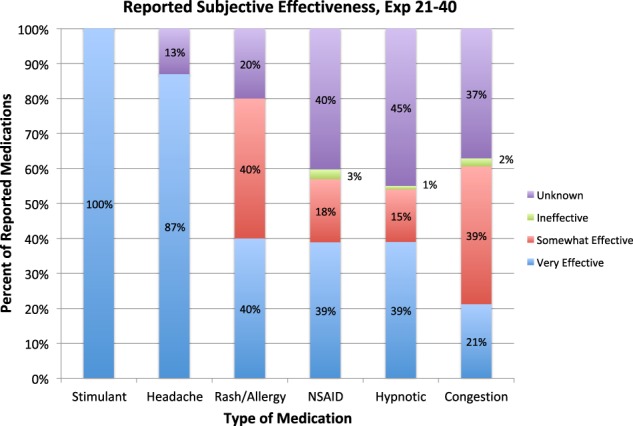
Subjective reports of medication effectiveness for six categories of clinical indications during ISS Expeditions 21–40.^2^ Medication use is grossly underreported due to the lack of a required or standardized documentation process. Reporting of drug consumption and any effectiveness or side effects is voluntary and anecdotal, unaccompanied by any study of metabolism, half-life, or peak concentration. Here, effectiveness is only indicated when crewmembers both reported drug consumption and subsequent perceived effectiveness. In many circumstances, logs of medication consumption do not record any information about effectiveness or symptom relief (noted here as “unknown“). NSAID non-steroidal anti-inflammatory drug

Careful review of medication reporting during flight suggests that medications may not be as effective in managing medical concerns as expected. During Expeditions 21–40, crewmembers did report that some medications were less effective than expected in managing common complaints (Fig. 2).^2^ Again, these data are limited by underreporting, but suggest that there may be some alteration of medication effect during spaceflight. In 1999, Putcha et al. published anecdotal reports of medication ineffectiveness during Space Shuttle flights, identifying 13 different medications that crewmembers reported as “not effective“ or “mildly effective“ in treating their symptoms, including oxymetazoline, zolpidem, flurazepam, aspirin, promethazine, temazepam, pseudoephedrine, acetaminophen, simethecone, bisacodyl, and the combination medications of promethazine/dextroamphetamine and phenylephrine/phenylpropanolamine.^5^ In 2014, Barger et al. published a discussion of sleep-medication use during Space Shuttle and ISS missions.^6^ According to this study, in 17–19% of cases where crewmembers took a sleep medication (zolpidem or zaleplon), a second dose was taken during the same night.^6^ This suggests that initial dosing may not have been as effective as desired, highlighting a potentially diminished response to medication during flight. However, these data are similarly limited by poor documentation, anecdotal and voluntary reporting, and difficulty in determining the timing of doses.

Unfortunately, these anecdotal reports of medication failure were not accompanied by study of drug metabolism. Given that flight surgeons are unable to truly examine their patients during flight, it is possible that crewmembers took medications (advised by their flight surgeons or otherwise) for an incorrect diagnosis or indication, which could account for the therapeutic “failure.“ For example, an astronaut may take a nasal decongestant for perceived sinus congestion attributed to air quality, but if in fact their feeling of congestion was caused by fluid shifts, any decongestant is unlikely to be effective. Thus, it is difficult to glean the true effect of spaceflight on drug performance.

## Medication stability

To be considered stable, a drug must maintain its physical and chemical properties over time. Pharmaceuticals can become unstable if any of these properties are altered, by time or environment. Alteration of physical properties includes changes in appearance or consistency; alteration of chemical properties includes loss of potency, alterations of dissolution and solubility of the medication, alteration of excipients (inert contents of a given formulation), excipient-active ingredient interactions, or toxic degradation.^7,8^ In order to determine that a pharmaceutical is unchanged by prolonged storage or environment, a drug must be demonstrated following exposure to have no significant alteration of its active pharmaceutical ingredients (APIs). At the same time, the drug must not develop significant degradation products that are either toxic themselves or in some way alter the pharmaceutical properties of the original medication.^9^ The United States Pharmacopeia (USP) provides guidelines for acceptable API content in medications approved by the U.S. Food and Drug Administration (FDA), commonly within 10% of label-specified content (though this can vary considerably by drug type or API).^10^ A medication would be considered degraded or unstable if API concentration fails to meet USP requirements following storage, environmental exposure, or time. Alterations of API or development of degradation products can affect drug potency and efficacy, rendering the drug ineffective or potentially dangerous.

One important consideration for pharmaceutical stability during long-duration spaceflight is the shelf life of flown drugs. The shelf life is determined by drug manufacturers and is expressed as an expiration date, after which time the manufacturer cannot guarantee the stability or potency of the medication. Shelf life of a given drug may vary among manufacturers given the intrinsic differences in formulation or inclusion of variable excipients, packaging, or other factors. A number of recent studies have addressed medication shelf life to determine whether terrestrial drugs can perform effectively beyond labeled expiration dates (Table 1). The Federal Shelf Life Extension Program (SLEP) was developed by the U.S. Department of Defense in conjunction with the FDA to defer the cost of drug waste by determining whether drugs can be used beyond expiration.^11^ In a 2006 study, Lyon et al. demonstrated that many medications, when maintained in original and unbroken packaging, may last significantly longer than labeled expiration dates, though stability was unpredictable and varied even between samples of the same drug, manufacturer, and drug lot.^11^ This study evaluated a number of medications currently flown in the ISS formulary. Similar studies by Matto et al.^12^ and Cantrell et al.^13^ presented further evidence of potential drug stability (when maintained in original and unbroken packaging) for longer time periods than expected (Table 1).

**Table 1:** Terrestrial U.S. evidence of drug stability beyond expiration for ISS formulary drugs

Drug	Dosage form	Lots tested	Mean extension (mo)	Extension range (mo)
*Lyons* et al.^11^				
Amoxicillin	Tablet*	21	23	21–23
Bupivicaine	Injectable solution	3	88	79–95
Ceftriaxone	Injectable powder	4	60	44–69
***Ciprofloxacin***	Tablet	242	55	12–142
Cimetidine	Tablet	242	55	12–142
Dexamethasone	Injectable solution	7	61	24–93
Diphenhydramine	Injectable solution	12	76	33–126
Doxycycline	Capsule	13	50	37–66
Guaifenesin	ER Tablet	7	85	39–122
Ketamine	Injectable solution	6	64	42–87
Meperidine	Injectable solution	6	89	32–128
Morphine	Injectable solution	13	89	35–119
Naloxone	Injectable solution	10	77	60–95
***Phenytoin***	Injectable solution	5	63	29–100
***Promethazine***	Injectable solution	9	51	28–73
*Cantrell* et al.^13^				
Acetaminophen**	Tablet			336–480
Caffeine**	Tablet			336–480
Caffeine**	Tablet			336–480
Hydrocodone*	Tablet			336–480
*Matto* et al.^12^				
Doxycycline	Tablet	3	179	144–204
Amoxicillin	Tablet	2	138	132–144

Medications presented were found by indicated studies to be stable beyond package expiration dates in terrestrial conditions. All drugs presented are in the ISS formulary; drugs marked with * are flown in a different formulation than that tested by the terrestrial study. Drugs marked by ** were compounded in a combination medication. Drugs in bold italics were found to be unstable after spaceflight in one or more spaceflight stability studies in contrast to terrestrial study results. The results extracted from Lyons et al.,^11^ Cantrell et al.,^13^ and Matto et al.;^12^ spaceflight stability results extracted from Du et al.,^15^ Wotring,^1^ and Wu et al.^17^

ER extended release

Additional work in the UK has also identified medication stability beyond labeled expiration dates. The electronic Medicines Compendium (eMC) contains current drug information from pharmaceutical companies licensed in the UK.^14^ Following publication of the results of the SLEP study, many of shelf lives of the eMC medications were revalidated by manufacturers and extended. In some cases, medications were approved for shelf lives of 3–6 years (Table 2). However, these changes have not been adopted by the U.S. FDA. Many of the eMC medications are developed by different manufacturing techniques than those in the U.S., with different excipients and packaging content. Regulation of the pharmaceutical industry in Europe differs from regulations in the U.S. Even so, the eMC may provide some additional evidence of shelf-life stability beyond labeled expiration.

**Table 2:** Terrestrial U.K. evidence of drug stability beyond expiration for ISS formulary drugs

Medication	Shelf life (mo)	Medication	Shelf life (mo)
***Acetaminophen*** tablet	36	***Loratadine*** tablet	36
Acetazolamide tablet	48	Medroxyprogesterone tablet	60
Amoxicillin capsule	36-48	***Melatonin*** tablet	36
Aspirin tablet	36	Metronidazole	36
Atropine injectable	36	*Modafinil tablet	36
***Azithromycin*** tablet	48–60	Mometasone nasal spray	36
Bisacodyl tablet	36	Naloxone injectable	36
Clindamycin capsule	36	Olopatadine ophthalmic solution	36
Clotrimazole cream	36	Omeprazole capsule	36
Diazepam injectable	36	Ondansetron tablet	36
Diphenhydramine tablet/injectable	36	Oxymetazoline nasal spray	36
Doxycycline capsule	36–60	***Promethazine*** tablet/injectable	36
***Fluconazole*** tablet	60	Pseudoephedrine	36
Hydrocortisone cream	60	***Sertraline*** tablet	60
***Ibuprofen*** tablet	36	Sodium chloride (normal saline)	36
Ketamine injectable	60	***Sulfamethoxazole/Trimethoprim*** tablet	60
***Levofloxacin*** tablet	36–60	Tamulosin capsule	48
***Lidocaine*** injectable	36	Triamcinolone cream	36
Lisinopril tablet	48	Valacyclovir tablet	36
*Loperamide capsule	60	*Zolpidem tablet	36

Medications presented were found by the United Kingdom’s electronic Medical Compendium (eMC) review to be stable for extended shelf life as indicated. All drugs presented are in the ISS formulary (though manufacturing, excipient content, and packaging may vary significantly between eMC formulations and spaceflight-flown medications). Drugs in bold italics were found to be unstable after spaceflight in one or more spaceflight stability studies in contrast to terrestrial study results. Drugs marked by an asterisk (*) were found to have degradant products of unknown significance in post-spaceflight analysis. The results extracted from the United Kingdom’s electronic Medicines Compendium;^14^ the spaceflight stability results extracted from Du et al.,^15^ Wotring,^1^ and Wu et al.^17^

Of the medications flown onboard the ISS, 87% have labeled shelf lives of less than 24 months. Thus, we are currently limited in the ability to provide an exploration pharmacy that extends beyond 24 months for the majority of medications flown today if we adhere to packaged expiration dates. SLEP and eMC evidence suggests that some medications may be able to be extended much further, potentially allowing therapeutic options beyond 24 months of spaceflight.

**Fig. 3:**
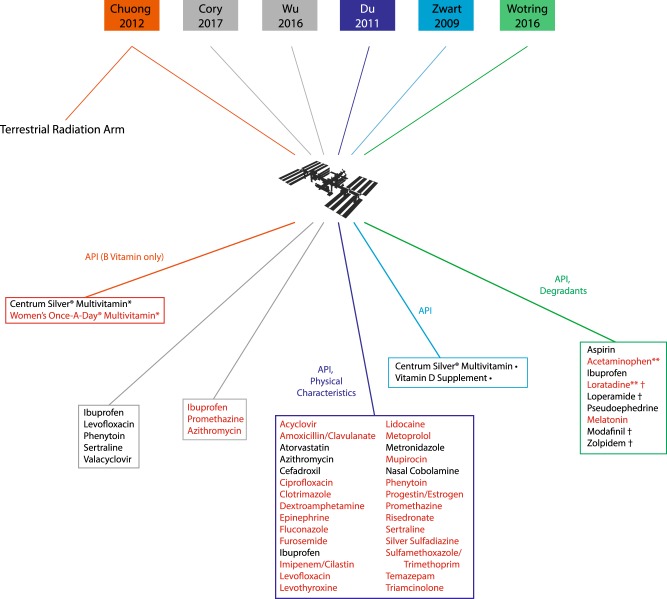
To date, there have been few studies of pharmaceuticals flown in the space environment. Du et al. (dark blue) compared active pharmaceutical ingredient (API) concentration and physical alterations in four flown payloads to ground controls.^15^ Wotring (green) identified degradation, impurity products, and API concentrations in an opportunistic sample of flown drugs without an available ground control.^1^ Zwart et al. (light blue) studied the vitamin content (API) of flown multivitamins compared to ground controls.^19^ Chuong et al. (orange) compared the API of B-complex vitamins in flown multivitamins, with ground controls available; a subset of these ground controls were irradiated with either hydrogen or iron ions at high dose and dose-rate dissimilar to the space environment.^18^ Finally, Cory et al. and Wu et al. (gray) analyzed an opportunistic sample of flown pharmaceuticals; these results have yet to be published, but early reports suggest variable results.^16,17^ Drugs highlighted in red were found to have alterations of API, physical characteristics, or contain significant concentrations of degradants or impurities after flight in one or more preparation of the indicated pharmaceutical. *Multivitamin preparations were analyzed only for B-complex API stability. **Drugs contained API concentrations within acceptable limits at time of study analysis, but would fail API analysis according to current standards. Drugs contained unspecified or unidentified impurity products of unknown significance. •Multivitamin content demonstrated time-related instability but showed no alteration specifically related to spaceflight exposure

However, limited data from stability studies of space-flown pharmaceuticals have offered evidence that some medications that demonstrate prolonged stability under terrestrial conditions may be altered by exposure to the space environment (Fig. 3). For example, Du et al.^15^ conducted a ground-controlled spaceflight study of API in flown drugs, demonstrating that amoxicillin–clavulanate, levofloxacin, trimethoprim, sulfamethoxazole, furosemide, and levothyroxine degraded before their expiration dates.^9,15^ An opportunistic sample of medications flown for > 550 days aboard the ISS also demonstrated degradation of pharmaceuticals after spaceflight.^1^ Wotring identified degradation and impurity products in aspirin, ibuprofen, loratadine, modafinil, and zolpidem.^1^ Du et al. also found alterations of physical appearance of some medications. Interestingly, some of these spaceflight-altered medications have been found to be stable during SLEP studies, such as ciprofloxacin, phenytoin, promethazine, and acetaminophen.

NASA directed additional recent studies by Cory et al.^16^ and Wu et al.^17^ to evaluate potency, purity, and drug degradation in pharmaceuticals flown onboard the ISS. These results have yet to be published, but in early reports, variable stability has been reported.^16,17^ Two additional studies examined the stability of multivitamin dietary supplements after spaceflight exposure. In both Chuong et al.^18^ and Zwart et al.,^19^ alterations of multivitamins over time were identified in both ground and flown samples when compared with time-zero controls, though neither study found convincing evidence of degradation specific to spaceflight-flown vitamins.^18,19^

Each of these studies has been severely limited by the ability to provide adequate ground control, control of confounders, or appropriate reproducibility given limited sample size and drug lot variability. As a result, it is unclear whether spaceflight-induced instability is common among pharmaceuticals and whether terrestrial stability study outcomes are applicable in the space environment. The impact of prolonged, low-dose radiation on pharmaceutical potency, toxicity, and degradation is not well understood.^2,15,20^ In addition to radiation, adjustments to drug packaging may play a role in altered drug stability during spaceflight. Many of the medications examined in spaceflight studies have previously been in operational use, meaning that packaging was opened at some time during flight; this may increase degradation secondary to factors, such as temperature, humidity, and radiation. Some of the pharmaceuticals currently flown onboard the ISS are repackaged by NASA pharmacists to manage mass and volume constraints and to limit packaging waste in the closed environment of a space vehicle. Repackaging itself may affect shelf life or stability of stored medications.^9,21^ Further information is needed to understand the role of the space environment and actions taken to prepare a medication for spaceflight, such as repackaging, on pharmaceutical stability.

## Spaceflight pharmacokinetics and pharmacodynamics

There is limited knowledge regarding alterations of pharmacokinetics (body effects on the drug, such as absorption, distribution, metabolism, and excretion of a medication) and pharmacodynamics (drug effects on the body) in the space environment. As the human body undergoes significant physiological and metabolic changes during spaceflight, it stands to reason that the effects of pharmaceuticals on an astronaut may change during flight.^9,22,23^ Alterations of hepatic blood flow due to fluid shifts from gravitational unloading^24–26^ may lead to altered hepatic metabolism and variable enzyme activity. Delayed gastric emptying due to microgravity conditions,^27^ associated space motion sickness,^28^ or side effects of medications used to control spaceflight-induced nausea^9^ may alter drug absorption in the gastrointestinal tract. Reduced total body water due to fluid shifting and renal excretion may alter the volume of distribution for consumed drugs.^29–33^ Variable protein expression, altered serum albumin levels, and altered renal blood flow may further affect drug absorption, distribution, metabolism, and excretion.^34,35^ However, research on the impact of spaceflight-induced physiological changes on pharmaceutical activity has largely been limited to observational reports and analog studies.^9,22^

**Fig. 4:**
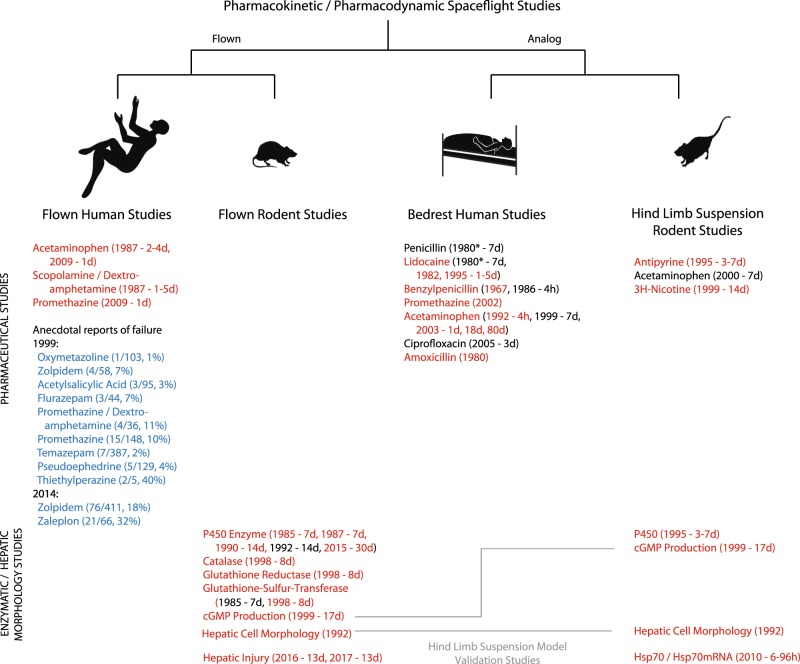
Pharmacokinetic and pharmacodynamic spaceflight and analog studies. Flown human studies, flown rodent studies, bedrest human analog studies, and hind-limb suspension rodent analog studies of PK/PD alterations in spaceflight. Drugs studied, publication dates, and duration (where available) are provided. Medications highlighted in red demonstrated significant alteration of PK/PD during spaceflight^36,37,39,40^ (or analog^48–57^). Medications highlighted in blue were anecdotally reported to be less effective during spaceflight compared to before or after spaceflight (percent of doses reported to be less effective is highlighted).^5,6^ Rodent studies of enzyme production or metabolism are similarly indicated. Enzymes highlighted in red demonstrated alteration in production or activity during flight^25,26,41–46^ (or analog^55,61–63^) compared with ground controls. Two validation studies were performed (gray lines) between flown rats and hind-limb suspension models,^24,47^ both suggesting poor correlation between spaceflight and the hind-limb suspension model. To date, there have been neither validation studies of the human bedrest model to human spaceflight PK/PD response nor have there been any validation studies comparing flown animal response to that of flown humans. cGMP cyclic guanosine monophosphate

Speaking generally, studies of PK/PD can be categorized as those of humans during spaceflight, animals during spaceflight, human analog studies (human bedrest), and animal analog studies (rodent hind-limb suspension). We will summarize cumulative understanding in each of these categories in the following sections (Fig. 4).

### Human flown studies

Few studies have evaluated PK/PD in actual astronauts before, during, or after flight. To date, flown studies have relied on salivary sampling rather than blood samples for drug concentration given the relative ease of salivary collection and storage. It is worth noting that salivary sampling has been identified as a flawed method of study for PK/PD given the unpredictable nature of drug crossover into saliva, and the fact that salivary concentration does not necessarily correlate to plasma concentration.^36,37^ Even so, salivary sampling has been the preferred (and only) method of studying PK/PD concerns during actual spaceflight.

In 1987, Cintron et al. performed a short observational series of salivary concentration of acetaminophen and scopolamine/dextroamphetamine during Space Shuttle flights.^36–38^ Each study observed 3–5 subjects via saliva collection after a single dose of medication before and during spaceflight. Early in flight, crewmembers demonstrated faster absorption, faster peak concentration, and more rapid drug clearance of either medication compared with ground observations. Later in flight, salivary concentrations after medication consumption demonstrated slower absorption and lower peak concentration compared with ground control. However, both studies were severely limited by high inter-subject variability and poor time consistency in sample collection. Salivary samples were collected at crewmember convenience, with numerous lost data points and inconsistent time periods for comparison. While data suggested the trends described above, minimal sample size and flawed experimental design limit any conclusions.^9,36,37^

A similar, unpublished NASA experiment performed in 2009 studied salivary promethazine concentrations after a single dose taken by crewmembers during spaceflight on mission day one of a Space Shuttle flight.^39^ Limited data available suggest higher peak concentration and shorter half-life of promethazine in saliva during the first day of spaceflight compared with ground data. Unfortunately, the results were never published or fully released. As with the studies by Cintron et al., salivary samples were collected at crewmember convenience with loose adherence to sample collection times, leading to numerous lost data points and difficulty in comparing flight data to ground controls. Finally, a 2009 Russian study of five crewmembers demonstrated decreased drug absorption rates and increased relative bioavailability for acetaminophen in tablet form during spaceflight.^40^ This study demonstrated altered pharmacokinetics based on drug formulation as well, identifying altered absorption time, elimination time, and volume of distribution between encapsulated and tablet formulations of acetaminophen.^40^ As with the earlier studies by Cintron et al., this study relied on salivary samples collected by on-orbit crewmembers with some lost data points, and the published report does not provide any timeline of when during the mission the data collection occurred. As physiological alterations induced by spaceflight can change over time in space, PK/PD early in flight may be quite different from PK/PD later in a mission; the absence of a mission timeline hinders the ability to draw conclusions from the Kovachevich et al.^40^ study.

### Animal flown studies

There have been a number of studies to evaluate enzymatic activity, concentration, or expression in animal models during spaceflight. Most studies are limited in sample size to 4–6 animals per experiment given mass, volume, and animal care constraints in spaceflight research platforms. In 1985, Hargrove and Jones studied the activity of the cytochrome P450 family of enzymes and of glutathione–sulfur-transferase (GST) in flown rats compared with ground controls.^41^ After 7 days of spaceflight, flown rats demonstrated a 50% decrease in P450 activity, but no change in GST activity. Similar follow-on studies by Merrill et al. showed variable alteration of P450 activity in flown animals.^26,42,43^ In 1987, after 7 days of spaceflight, Merrill et al. demonstrated a 50% reduction in P450 activity in six flown rats,^43^ whereas in 1990 the same research team demonstrated a reduction in P450 activity of only 15% in five rats after 14 days of spaceflight.^42^ In 1992, Merrill et al. again studied P450 activity in rats after 14 days of flight, this time finding no significant difference in enzyme activity in flown rats compared with ground controls.^26^ A further study in 1998 regarding enzyme expression after 8 days of spaceflight identified decreased concentration of catalase, glutathione reductase, and GST in flown rats compared with ground controls, but did not examine enzyme activity.^25^ More recently, Moskaleva et al. demonstrated a significant increase in P450 enzyme concentration after 30 days of spaceflight in four flown mice.^44^ Other recent studies have identified altered enzyme activity, such as increased lipotoxic activation^45^ and increased autophagy and proteasome activity,^46^ in flown mice.

While these studies suggest that there may be alterations of enzyme expression, concentration, and activity during and after spaceflight, it is difficult to interpret the results that vary so widely between experiments of variable design and intent. Additional confounding factors of time of exposure to spaceflight, environmental conditions, light–dark cycling, or time to post-flight recovery and sampling may further alter the results and limit the ability to compare findings between studies. Moreover, there has been no validation of rodent models with respect to extrapolation of human response during spaceflight. There have been two studies that attempted to validate terrestrial rodent analog studies that use hind-limb suspension to mimic microgravity-induced fluid shifts by comparing analog the results to those in flown animals. In 1992, Racine et al. studied hepatic cellular morphology in flown and suspended rats, demonstrating alterations in morphology in both research arms compared with non-suspended ground controls.^24^ Flown rats demonstrated larger hepatic cells, increased glycogen and lipid storage, and decreased presence of Kupffer defense cells compared with suspended animals, suggesting that hind-limb suspension does not adequately simulate microgravity for hepatic studies. Similarly, Carcenac et al. studied cyclic guanosine monophosphate (cGMP) production in rats flown for 17 days to enzyme production in hind-limb suspension analog rodents.^47^ Both research arms demonstrated significant differences in cGMP production compared with ground controls, but alterations varied widely between flown and suspended animals. As with the Racine et al.^24^ study, these results suggest that hind-limb suspension is a poor model for the complex metabolic effects of spaceflight.

### Human bedrest analog studies

Human bedrest is a commonly studied terrestrial analog to spaceflight due to similarities in body fluid shifts due to microgravity or head-down positioning. A number of human bedrest studies have sought to identify alterations of drug metabolism due to postural changes. Despite these studies, there has been no common trend regarding absorption, distribution, metabolism, or elimination of medications during bedrest. This may be due to variability in study design, including highly variable time frames (lasting minutes to hours to months), limited subject pools, adherence to bedrest positioning, or comparability of study variables.

Many bedrest studies have addressed metabolism of antibiotics, an important topic when considering appropriate medications to include in a spaceflight pharmacy. One of the earliest studies of head-down bedrest alterations of metabolism was performed by Levy et al. in 1967, in which intramuscular administration of benzylpenicillin was followed by increased renal clearance and decreased metabolic degradation in head-down bedrest subjects compared with normal controls.^48^ In 1980 Kates et al. demonstrated no change in absorption, distribution, clearance, or half-life of intravenously administered penicillin in bedrest subjects compared with normal controls.^49^ This study involved horizontal positioning and not head-down tilt, thereby limiting conclusions regarding head-ward fluid redistribution as seen in microgravity. A further study by Rumble et al. in 1986 demonstrated no change in absorption, distribution, clearance, or half-life of benzylpenicillin in bedrest subjects compared with controls.^50^ Additional antibiotic studies have included Roberts et al.,^51^ who identified a decreased peak serum concentration and faster time to peak after administration of amoxicillin in bedrest subjects, and Schuck et al.,^52^ who identified no change in ciprofloxacin serum concentration in bedrest subjects after 3 days of recumbency.

Numerous studies have addressed the metabolism of acetaminophen in bedrest, including Renwick et al.,^53^ who found an increased time to maximum acetaminophen concentration after 4 h of left lateral recumbency, Rumble et al.,^54^ who demonstrated no change in absorption or elimination of acetaminophen after 7 days of bedrest (though, notably, subjects were allowed to ambulate every morning of the study), and Gandia et al.,^55^ who found no change in acetaminophen absorption after 1 day, 18 days, or 80 days of bedrest. Additional studies have addressed lidocaine metabolism during bedrest. The 1980 Kates et al.^49^ study found no change in absorption, distribution, clearance, or half-life of lidocaine in bedrest subjects compared with normal controls (though again, subjects were supine and not head down). Saivin et al.^56^ identified increased lidocaine clearance after 4 days of head-down bedrest, and Feely et al.^57^ found evidence of increased lidocaine clearance immediately after reclined positioning, suggesting altered clearance may be due to instantaneous alterations of hepatic blood flow, as affected by postural changes.

It is difficult to identify a common theme in these results. Indeed, due to numerous study variables, overall results range from inconclusive to directly contradictory. Furthermore, it is worth noting that there are no studies to validate PK/PD alterations in bedrest as an appropriate analog to spaceflight. While bedrest-induced physiological changes may mimic microgravity with regard to fluid shifting, fluid distribution, and hydration status do not necessarily match those of spaceflight.^31,58–60^ Certainly, bedrest does not mimic the stress or metabolic demands of a typical crew workday aboard the ISS. Thus, bedrest results are limited by both inconsistent evidence and inadequate modeling of the spaceflight environment.

### Rodent hind-limb suspension analog studies

A common animal ground analog for spaceflight is that of hind-limb suspension, where rodents are suspended by their tail to induce a head-ward fluid shift mimicking that of microgravity. Similar to human bedrest, fluid shifting theoretically mimics that of spaceflight, but in reality, there are concerns about the accuracy of this analog. As described above, two validation studies comparing suspended rats to those exposed to short-duration spaceflight demonstrated different alterations of hepatic cellular morphology and enzyme expression between the two rodent groups.^24,47^ Even so, the rare and limited opportunities to perform spaceflight research has driven researchers to make use of even this flawed analog for PK/PD studies.

Suspension studies have included those addressing enzyme expression and concentration, as well as metabolism of specific medications. In 1995, Brunner et al.^61^ suspended rats for 3–7 days and demonstrated alterations of serum albumin concentration, antipyrine clearance and concentration, and concentration of enzymes in the cytochrome P450 family compared with control (non-suspended) rats. In 1999, Chowdhury et al.^62^ demonstrated increased elimination of nicotine after 14 days of rodent suspension, though authors postulated that this might be due to animal stress response related to the suspension technique. In 2000, Brunner et al.^63^ demonstrated no change in acetaminophen concentration or half-life in suspended rats compared with control animals after 7 days, though a few years later Gandia et al.^55^ found increased absorption of acetaminophen and decreased volume of distribution after 1, 2, and 5 days of suspension compared with control animals. More recently, hind-limb suspension was found to increase expression of hepatic enzyme Hsp70 and Hsp70 messenger ribonucleic acid (mRNA) in rats, which authors Cui et al. attribute to stress response.^64^

As with bedrest studies, the results suggest that spaceflight (or analog environments) may alter metabolism or drug response. However, the results of rodent hind-limb suspension models are often contradictory and difficult to interpret given the numerous confounders and poor correlation between suspension and spaceflight rodent responses.

## Additional considerations

Just as humans may respond differently to pharmaceuticals in the spaceflight environment, pathogens may be altered as well. When developing an appropriate formulary for exploration spaceflight missions, it will be important to identify appropriate antibiotics for the treatment of a variety of potential infections. Antibiotics will be subject to the same shelf life and PK/PD concerns that have been described in previous sections of this article, but there are additional concerns regarding microbial response to antibiotics in the space environment that make antibiotic choice particularly challenging.

Spaceflight is known to have negative effects on human immune response. A comprehensive review on immune and microbiome alterations associated with spaceflight was recently published by Taylor;^65^ in brief, prolonged spaceflight is associated with decreased response of neutrophils, macrophages, lymphocytes, and natural killer (NK) cells to stimuli.^66–68^ In addition, studies have demonstrated a reduction in beneficial microbial species from crew gastrointestinal tracts,^69–71^ which may place crew at the risk for increased susceptibility to infection.

In 1985, Tixador et al. demonstrated increased antibiotic resistance of spaceflight-cultured *Staphylococcus aureus* and *Escherichia coli* to antibiotics, including kanamycin, colistin, erythromycin, chloramphenicol, and oxacillin.^72,73^ A similar study by Juergensmeyer et al. again identified resistance of spaceflight-cultured *Staphylococcus aureus* to numerous antibiotics, but demonstrated normal-to-increased susceptibility of other organisms to antibiotic exposures.^74^ A study by Kacena et al. demonstrated heavier growth of spaceflight bacterial cultures compared with ground controls, suggesting that microgravity may increase bacterial replication (though colonies were susceptible to normal antibiotic exposures).^75^

In addition, studies of yeast growth have demonstrated increased mutation rates during spaceflight, perhaps due to increased radiation exposure,^76^ a factor that will be compounded by missions outside of LEO and away from the protective shielding of the Earth’s magnetosphere. Other studies have found increased metabolic activity, increased biomass and biofilm production, and increased metabolite production of bacterial cultures in spaceflight.^65,77–79^ Spaceflight-grown biofilms demonstrate altered structure and increased complexity compared with ground controls.^80,81^ The combination of altered microbiological growth, poor antimicrobial response, and decreased immune system function may place crew at increased risk for infection.

Taken as a whole, these studies provide preliminary evidence that bacteria may experience enhanced and altered growth potential and some degree of antimicrobial resistance during spaceflight. While these studies are far from comprehensive, they do suggest that much more research is needed to understand the growth potential, virulence, and resistance to treatment of bacteria and associated bacterial infection during spaceflight. These factors are particularly concerning when considered in the context of exploration spaceflight where mission duration and exposures for crews are estimated to last up to 3 years. In this context, the compounded effects of spaceflight-induced alterations of human immune response, the limited understanding of PK/PD during flight, and the constraints of shelf life and instability of medications present unknown risks beyond our current understanding of spaceflight in LEO.

Finally, selection of an appropriate pharmacy must take into consideration the potential for altered medication response specific to individual crewmembers due to genetic polymorphisms. Pharmaceutical interventions tailored through use of pharmacogenetics and pharmacogenomics have the potential to reduce formulary mass by optimizing drug selection for astronauts. As an example, NASA currently empirically identifies crewmember susceptibility and response to various medications for sleep and alertness prior to flight and includes crew-preferred medications and dosing guidelines for use during a given mission.^82^ With new evidence identifying genetic variability in sleep requirements,^83^ countermeasures for sleep may further drive personalization of sleep medications in a future exploration pharmacy.

The fields of pharmacogenetics and pharmacogenomics are developing rapidly, as are their potential applications to spaceflight, with new research advances addressing genetic- and population-based differences in drug response, as well as sex- or age-specific drug susceptibility.^4^ The drive to personalize medication choices for future mission may rapidly increase the number of medications that must be studied for spaceflight applications. As each new medication may have a different stability or PK/PD profile in the space environment, each additional drug that may be desirable for formulary inclusion may increase the need for controlled research studies, packaging analysis, and safety profiling prior to approval for exploration spaceflight. Personalized preference for a medication in a terrestrial environment may not translate to spaceflight effectiveness due to alterations of metabolism in flight or the stability of the drug of choice. As NASA intends to fly increasingly distant missions to the moon and Mars in the next decades of spaceflight, the need to expand spaceflight pharmaceutical knowledge is driven by projected mission dates and by the continued availability of the ISS as a laboratory for investigating these effects.

## Discussion

Pharmaceutical intervention is an essential component of risk management planning for astronaut healthcare during exploration spaceflight. However, there is limited understanding of current spaceflight pharmaceutical usage and effectiveness, drug stability, and altered PK/PD in the space environment. This limitation significantly constrains efforts to assemble an exploration formulary that is both comprehensive enough to prevent and treat anticipated medical events and chemically stable, safe, and robust enough to last the duration of the mission.

The majority of operational interventions that address medical risks in spaceflight are likely to rely on pharmaceutical use. A current review of NASA’s Human Research Roadmap identifies medication use as the primary countermeasure for prevention or management of most conditions, including Spaceflight-Associated Neuro-ocular Syndrome (SANS), cognitive/behavioral conditions, acute infections, and degenerative tissue risks.^84^ In an analysis of medical capabilities needed for a Mars transit mission, NASA’s Exploration Medical Capability Element (ExMC) has preliminarily identified onboard medications as the largest single component of the complete medical capability, potentially accounting for more than a quarter of all medical interventions. For perspective, medications may account for a larger proportion of the medical capability tradespace than procedures, surgical intervention, diagnostic imagery, and examinations combined.^85^ This projection is consistent with our limited experience on ISS. Medical kits aboard the ISS contain medications and supplies to help crewmembers cope with a variety of possible medical events, including space motion sickness, sleep disruption, illnesses, injuries, and behavioral health problems.^86^ While medication-usage reporting is not consistent, historical documentation indicates that more than 80% of crewmembers took medications at some time during Space Shuttle missions,^87^ further demonstrating our reliance upon pharmaceuticals to manage onboard health concerns.

With the inability to resupply, any exploration mission must be capable of providing a robust pharmaceutical formulary that is stable enough to last throughout the mission, comprehensive enough to treat all potential medical events, and safe and effective, despite known alterations of metabolism that occur during spaceflight. This becomes a challenge given the numerous limitations to our understanding of pharmaceutical stability and PK/PD during spaceflight. As a result, it is difficult to identify appropriate medications for inclusion in a spaceflight pharmacy, particularly for longer missions that venture outside of LEO. NASA’s Human Systems Risk Board has recently highlighted this challenge by recognizing pharmaceutical concerns for long-duration, exploration spaceflight as a high-level risk.^2^ NASA’s ExMC has identified our current inability to provide a safe and effective pharmacy for exploration spaceflight as a major research gap.^4^ The ExMC Pharmacy Research Team recently developed a comprehensive research plan to help inform and address some of the issues presented here.^88^ Even so, approaching mission and vehicle design-freeze deadlines and the pressing need to identify and declare a planned formulary for near-future missions are threatening to outpace even the most rapidly achievable drug studies.

To fully understand the issue of drug stability for long-duration spaceflight, studies would need to examine shelf-life extension in the space environment with a particular focus on space radiation exposure and the impact of altered packaging for vehicle constraints. Such studies could be accomplished using the ISS or additional vehicles as research platforms, particularly if appropriate ground controls were established. As the ISS is intended for decommissioning or commercialization within the next decade, studies that intend to use this invaluable research platform would need to be initiated rapidly. Inclusion of pharmaceuticals onboard future unmanned vehicles, particularly those intended for missions outside of LEO, may also provide much-needed data; however, most of these missions do not intend to return payload to Earth, thereby limiting analysis options to studies that can be completed remotely. Additional validation of terrestrial radiation beam exposures as comparable or translatable to spaceflight radiation exposure could allow for more rapid terrestrial evaluation of pharmaceuticals without the expense or time constraints of spaceflight studies.^2^

Given the poor correlation between spaceflight and analog models and the lack of validation of animal studies of PK/PD, the highest-yield studies of PK/PD would be those performed on human subjects during spaceflight. Utilization of the ISS as a research platform, with well-designed and controlled studies of human response to medication use, could provide much-needed understanding of PK/PD during spaceflight. At the same time, there are numerous limitations to this approach. First, subject pools are limited by the vehicle and mission constraints; at any given time, there are 3–6 astronauts and cosmonauts living aboard the ISS. Until future vehicle capabilities are realized, human presence in space will be limited to very small sample size, with any studies requiring prolonged time periods to accumulate generalizable and deidentifiable data for analysis. In addition, pharmaceuticals administered for research purposes alone will be ethically limited, as exposure to unnecessary medications has the potential to cause astronauts harm or affect their ability to perform operational tasks necessary for mission success. As a result, many human spaceflight studies may be limited to collection of data on medications used by crewmembers under medically indicated circumstances. Even so, these data would greatly advance our understanding of medication usage, side effects, and effectiveness if collected in a more comprehensive manner than historical methods. Use of medication-tracking software and thorough, prompted queries regarding crewmember medication use and response would provide valuable insight into the therapeutic effectiveness and volume of medications required for future missions. International collaboration to increase the number of subjects would be ideal.

Additional studies of PK/PD in animals may provide some insight, though models need validation before extrapolating results to humans. Given mass, volume, and animal care constraints in flight, rodents may continue to be the most opportune animal model for future drug studies. If so, careful and controlled studies of drug absorption, distribution, metabolism, and excretion in rodents should be validated through comparison of terrestrial animal and human response. Where possible, studies should be further correlated to spaceflight human response through comparative analysis of known human medication response (such as through medication-tracking software, as described above) or any ethically feasible study of human drug metabolism via analysis of serum or salivary drug concentration after pharmaceutical use during spaceflight.

The limitations in our understanding of pharmaceutical use and effectiveness have been manageable, while human spaceflight has remained in LEO. In the exploration paradigm, resupply will not be available and mission length will expand significantly. In all likelihood, pharmaceuticals will become increasingly important in these extended missions just as our ability to provide a safe and effective pharmacy becomes curtailed. There is broad uncertainty surrounding the compound effects of the spaceflight environment, including chronic radiation exposure, long-term isolation, extended microgravity exposure, increasing risk of medical and psychological challenges, and decreasing resources. This drives a need to better understand the complexity of this problem and the contributing factors that are within our ability to control. Pharmaceutical use by crews, medication stability in the spaceflight environment, and PK/PD are all challenges that can be reasonably characterized within the remaining lifetime of the ISS. Well-planned research efforts that provide actual flight data when available as well as translational studies of comparative effects of flown animal metabolism or terrestrial analogs could greatly improve upon our current understanding. Careful study of pharmaceutical use and response during current spaceflight activities, with comprehensive tracking of effectiveness and side effects, could provide much-needed understanding of pharmaceutical success and failure. By committing to a strategic path forward to address this challenge, NASA has the opportunity to make significant strides toward the realization of a safe and effective pharmacy that can support future exploration spaceflight.

### Reporting summary

Further information on experimental design is available in the Nature Research Reporting Summary linked to this paper.

## Supplementary information

Reporting Summary Checklist

## Data Availability

All data in this paper can be found in previous publications cited within the text.

## Supplementary information

**Supplementary information** accompanies the paper on the *npj Microgravity* website (10.1038/s41526-019-0075-2).
